# Meta-analyzing the efficacy of 3D printed models in anatomy education

**DOI:** 10.3389/fbioe.2023.1117555

**Published:** 2023-02-20

**Authors:** Zhen Ye, Hanming Jiang, Suyun Bai, Tao Wang, Duxiao Yang, Haifeng Hou, Yuanying Zhang, Shuying Yi

**Affiliations:** ^1^ School of clinical and basic medicine, The Second Affiliated Hospital of Shandong First Medical University, Tai’an, China; ^2^ School of Public Health, Shandong First Medical University and Shandong Academy of Medical Sciences, Tai’an, China

**Keywords:** three-dimensional printing (3D printing), meta regression, post-test, satisfaction, anatomy education

## Abstract

Three-dimensional printing models (3DPs) have been widely used in medical anatomy training. However, the 3DPs evaluation results differ depending on such factors as the training objects, experimental design, organ parts, and test content. Thus, this systematic evaluation was carried out to better understand the role of 3DPs in different populations and different experimental designs. Controlled (CON) studies of 3DPs were retrieved from PubMed and Web of Science databases, where the participants were medical students or residents. The teaching content is the anatomical knowledge of human organs. One evaluation indicator is the mastery of anatomical knowledge after training, and the other is the satisfaction of participants with 3DPs. On the whole, the performance of the 3DPs group was higher than that of the CON group; however, there was no statistical difference in the resident subgroup, and there was no statistical difference for 3DPs vs. 3D visual imaging (3DI). In terms of satisfaction rate, the summary data showed that the difference between the 3DPs group (83.6%) vs. the CON group (69.6%) (binary variable) was not statistically significant, with *p* > 0.05. 3DPs has a positive effect on anatomy teaching, although there are no statistical differences in the performance tests of individual subgroups; participants generally had good evaluations and satisfaction with 3DPs. 3DPs still faces challenges in production cost, raw material source, authenticity, durability, etc. The future of 3D-printing-model-assisted anatomy teaching is worthy of expectation.

## 1 Introduction

Three-dimensional (3D) bioprinting is a rapid prototyping technology that can print various tissues and organs by assembling biological materials in a layer-by-layer manner under the control (CON) of a computer-aided design model (Song et al., 2021). Commonly used printing materials include acrylonitrile—butadiene—styrene, polypropylene, natural polylactic acid, (Yanar et al., 2018), Laser-induced graphene, (Luong et al., 2018), and resin, (Sutton et al., 2018). New printing materials such as polyether ether ketone are used to produce samples with tensile strength that exceeds 200% that of traditional materials such as acrylonitrile-butadiene-styrene (Gordelier et al., 2019). Common 3D printing technologies include stereoscopic lithography, selective laser sintering, melt deposition modeling, and inkjet 3D printing (Cheng et al., 2021). Melt deposition modeling printers are now the most popular 3D printers on the market (Gordelier et al., 2019). In the past decade, 3D bioprint technology has developed rapidly, finding a place in the aviation, defense, automotive, forensic, dental, prosthetics, and other industries as well as in surgery (Deshmane et al., 2021; Zhang et al., 2021; Pavan Kalyan & Kumar, 2022). 3D printing (3DP) technology is profoundly impacting on biomedicine, as three-dimensional printing has been used to label cells, reproduce tissue or an entire organ, and make surgical replicas for prospective surgical guidance, consultation, and medical education (Cacciamani et al., 2019).

Due to the modernization of the medical curriculum and the advancement of technology, medical anatomy education has gone beyond mere autopsy. Plasticizing technology and 3DP technology are gaining importance (Chandrasekaran et al., 2021), and with the progress of science and technology, 3DP has gradually entered medical education (Ye et al., 2020). For example, 3D-printed anatomical models can replace cadavers or human anatomical structures as teaching tools for medical students or young clinical doctors, as real cadavers have many deficiencies in practical medical teaching. For example, to prevent decomposition, a formalin immersion is required, which is often too harsh for allergic students. There are also limits to the supply of bodies.

There are several lines of research in previous CON studies of three-dimensional printing models (3DPs) in anatomy teaching. The most common CON experiment tests the effects of 3DPs vs. traditional teaching methods using traditional teaching tools, such as two-dimensional (2D) pictures, books, photo albums, and CT films (Al-Badri et al., 2022; Nicot et al., 2022; O'Brien et al., 2021). Another common comparative study involves 3DPs and traditional teaching aids vs. solely traditional teaching aids (Karsenty et al., 2021; Hu et al., 2022; Lau & Sun, 2022). A relatively rare grouping method is a CON crossover study: Each group experiences 3DP model and traditional teaching aids, but the time sequence is different (Mogali et al., 2021). There are two main types of participants in these CON trials, medical students in medical colleges and residents in hospitals; studies relatively rarely include unidentified temporary recruiters or senior doctors in hospitals. CON experimental studies of medical students or residents using 3DPs mainly examine two aspects: anatomical knowledge and training in surgery or the use of instruments, and similarly, there are two main types of evaluation of training results: test results after training, and the feedback of participants on such factors as satisfaction, learning confidence, and usefulness. We posit that the feedback of trainees after training, whether regarding satisfaction, usefulness, helpfulness, or the acquisition of knowledge, can serve as an index of overall satisfaction with 3DPs. Therefore, in this study, we focus on the test results after training and the reported satisfaction of students.

3D printed models are widely used in medicine. Common applications include training doctors in surgery and medical diagnosis, and anatomy training for medical students and residents as a means to help students enhance their knowledge of anatomy. Results of 3DP in different randomized CON studies are not completely consistent due to differences in test content and test populations. For example, Cai’s study found that compared with the CON group, the 3DP group had better test scores (Cai et al., 2019); however, Hu did not find a statistical difference (Hu et al., 2022). In this study, we focused on the role of 3D printed models in helping medical students and residents with anatomical knowledge. In the past, our group and other research groups have published meta-analysis of the role of 3D printed models in anatomical learning (Lau & Sun, 2019; Fleming et al., 2020; Ye et al., 2020). However, a previously published meta-analysis of 3D printing models showed the following deficiencies: 1. The CON and experimental groups were roughly divided (Ye et al., 2020); 2. because of the inconsistency in different research scoring standards and test questions, the mean difference might not be suitable for continuous variables, so standardized mean difference (SMD) should be used to combine data (Lau & Sun, 2019); and 3. fewer CON trials were included (Fleming et al., 2020; Pearce et al., 2022; Salazar et al., 2022), and some lacked quality assessment of the included research literature. Compared with previous meta-analysis studies, our study has the following characteristics or strengths: 1. The largest number of studies has been included; 2. The literature search was more methodical, i.e., we focused on the role of 3D models in assisting anatomical knowledge, and therefore included both medical students and residents in the study; 3. Grouping is more reasonable and detailed. For example, some CON trials were 3DPs vs. 2D, while other CON trials were 3DPs + CON vs. CON, which need to be treated separately; and 4. Regression analysis is used to explore the factors that influence the results.

Through this meta-analysis, the role and significance of 3DPs for medical students and residents to learn human anatomy were expanded, and basic work regarding the future application of a 3DPs model in anatomy teaching in medical colleges and hospitals was performed.

## 2 Materials and methods

We followed the PRISMA guidelines (https://www.equator-network.org/reporting.-guidelines/prisma/) in this study.

### 2.1 Searches

**FIGURE 1:**
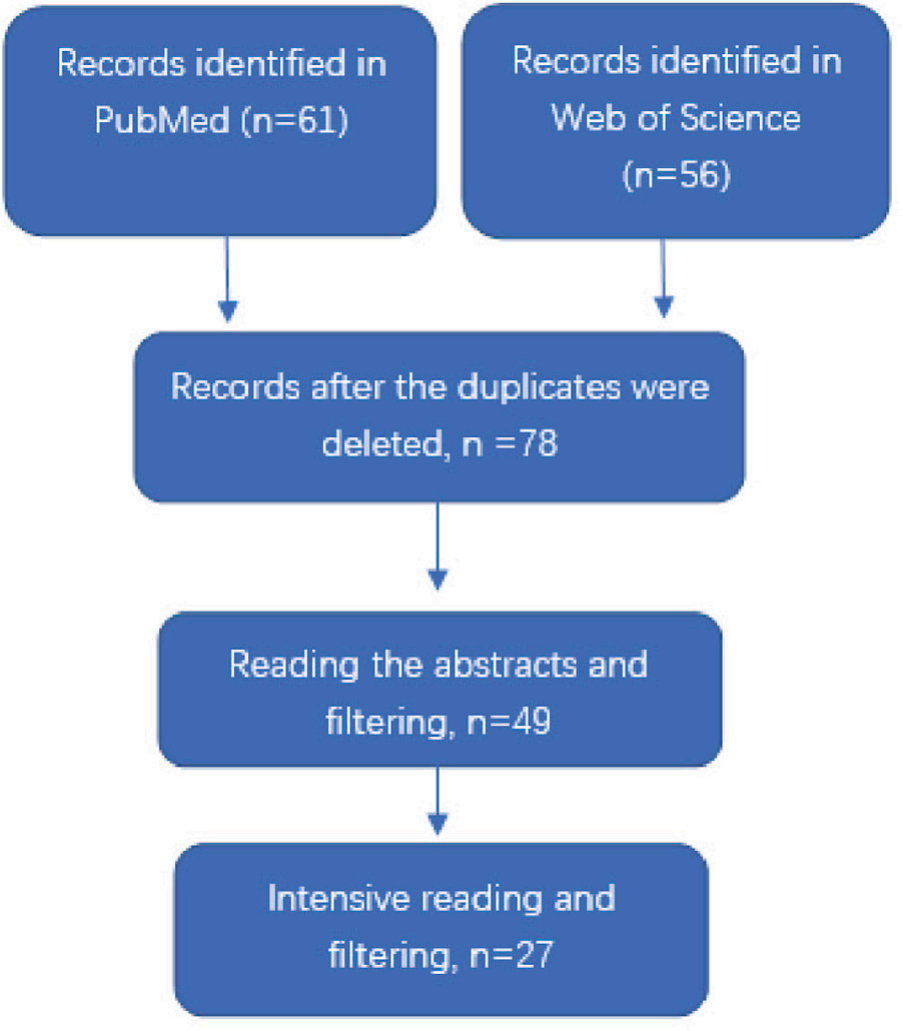
Flowchart of selection and screening of the studies.

We used two databases, PubMed and Web of Science, to retrieve documents. The set search keywords were (3D printed model or Three-dimensional printing model) and (medical or medicine) and (education or teaching) and (students or residents) and (group or study) and anatomy and p. The language of the literature was limited to English, and literature was retrieved until June 2022. Inclusion criteria of literature was: 1. A comparative study of anatomy teaching of human organs; 2. The research site was a medical college or hospital; 3. The subjects were medical school students or hospital residents; 4. The aim of the study was to evaluate the effect of 3D printed models on anatomy teaching of human organs; and 5. The results included testing of anatomical knowledge of human organs using 3D printed models and feedback from participants. Exclusion criteria for literature was: 1. Documentation of how 3DPs are made; 2. Literature on 3D-printed animal models; 3. There was no CON group, or the 3DPs group and the CON group were cross-studied; 4. Previous reviews, case reports, meta-analyses; and 5. Studies from which valid data cannot be extracted. The retrieval flow chart was presented in Figure 1.

### 2.2 Data extraction

The two authors discussed the extraction of literature data, and resolved disputes through negotiation. The contents of literature extraction included publication time, name of the first author, regional source of the study, sizes of the experimental group and CON group, teaching tools used by the CON group, and results of analysis and comparison. Two types of analysis results were extracted, the performance test after training and the satisfaction feedback of the participants after training. Studies were roughly screened by reading the abstracts, then fine screening of the literature was conducted through reading the full texts.

### 2.3 Quality assessment of included studies

Quality of the included studies was assessed using the tools from the National Institute of CON Intervention Studies. Two researchers evaluated the quality of the literature included in the study using the Study Quality Assessment Tools developed by the National Heart, Lung, and Blood Institute (www.nhlbi.nih.gov/health-topics/study-quality-assessment-tools). Evaluation items included whether methods adopted were random or blind, whether baseline was similar, and so forth, for a total of 14 items. Each item was evaluated using the following three options: yes, no, or unclear. If “yes” was used for ≥50% of items, the quality was assumed to be good; ≥30%, the quality was fair, and <30%, the quality was poor.

### 2.4 Meta-analyses

The variation in different studies in systematic review is called heterogeneity. If the *p*-value of the heterogeneity test is <0.1, it is considered to be statistically significant, and a random effects model is generally selected for combined data. If *p* ≥ 0.1, it is considered not to be statistically significant, and a fixed effects model can be selected. For the experimental group and CON group, the results were scored using continuous variable analysis and compared using a SMD. Since different CON tests adopt different standards for test scores, the SMD method was used here. The Inverse variance method was used for the statistical analysis of continuous variable data. Relevant usage of “metacont” function of R language used: y1 = metacont (n.e,mean.e,sd.e,n.c,mean.c,sd.c,studlab, data = data).

n.e: Number of observations in 3DPs group.

mean.e: Estimated mean in 3DPs group.

sd.e: Standard deviation in 3DPs group.

n.c: Number of observations in CON group.

mean.c: Estimated mean in CON group.

sd.c: Standard deviation in CON group.

The result was a comparison of rates using a dichotomous variable analysis through which Risk Ratio (RR) values were calculated for the event incidence of the experimental group compared to the event incidence of the CON group. The Mantel-Haenszel method was used for statistical analysis of dichotomous variables. The “metabin” function of R language was used:

y2 = metabin (event.e,n.e,event.c,n.c,studlab, data = data).

event.e: Number of events in 3DPs group.

n.e: Number of observations in 3DPs group.

event.c: Number of events in CON group.

n.c: Number of observations in CON group.

The experimental group represented the group that applied 3DPs. The CON group was given the usual teaching tools, for example, 2D, 3DI, or physical models. The 2D group included 2D imaging, CT, MRI images, atlases, and slides. Physical models included commercial models made of different materials, plastic models, and real cadaver specimens. We compared the test scores of the CON group and the 3DPs group after training and learning. We also compared the satisfaction of the participants in the CON group with those in 3DPs. The comparison of satisfaction falls into two broad categories. One is the score of satisfaction, which belongs to the continuity variable, and the other is the percentage of satisfaction, which belongs to the two classification variables. We used a combination of continuous variables and binary data, respectively.

### 2.5 Sensitivity and risk of bias

As the number of merged CON studies was greater than four, sensitivity and publication bias were analyzed. Sensitivity was detected using the leave-one-out method. Classic generic tests for funnel plot asymmetry, based on rank correlation or linear regression method, were applied, specifically the “Begg” and “Egger” tests. The “Begg” test is based on the rank correlation between standardized treatment estimates and variance estimates of estimated treatment effects (Begg & Mazumdar, 1994), while the “Egger” test is based on a weighted linear regression of the treatment effect on its standard error (Ribero et al., 2015). The function usage for detecting funnel symmetry was as follows:

y3 = metabias (x, method. bias = “Egger”, k. min = 5);

y4 = metabias (x, method. bias = “Begg”, k. min = 5);

x: An object of class meta or estimated treatment effect in individual studies.

### 2.6 Regression analysis

If more than 8 CON studies were included in the meta-analysis and no fewer than 4 studies were included in the subgroup, meta-regression was conducted to explore potential factors affecting the analysis results. A bubble plot was used to display the result of the meta-regression, which is a scatter plot with the SMD value for each study on the *y*-axis and the covariate used (for example, the time and country of publication) in the meta-regression on the *x*-axis.

Function usage of meta regression: y5 = metareg(x).

### 2.7 Analysis tools and statistical analysis

R4.11 (https://www.r-project.org/) was used for data meta-analysis. The data package used was the “meta” package. Microsoft Excel 2016 was used for data management. Alluvial diagram drawing was conducted using online software production (https://app.rawgraphs.io/). *p*-values less than 0.05 were defined as statistically significant.

## 3 Results

### 3.1 General characteristics of the included articles

**FIGURE 2:**
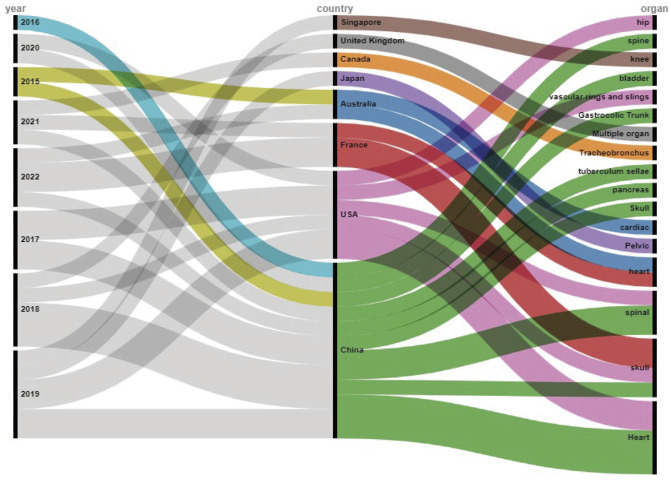
Alluvial diagram of the 27 articles included in this meta-analysis.

We included 27 articles published from 2015 to 2022 in the study (Figure 2; Table 1). Among them, six articles were from 2019—the largest number, followed by five in 2018. All articles came from eight countries, with 12 from China (maximum), followed by five from the United States. The 27 articles involved 15 human anatomical sites, of which the heart was the subject of the most articles (seven), followed by the skull with five articles. Among the 27 studies, 11 were of good quality, and 16 were of fair quality. Three studies that came from France, were all rated as good (Table 2).

**TABLE 1:** Basic information of all 27 studies included in the meta-analysis.

Year	Author	Country	Organ	Control	Intervention
2022	Al-Badri	France	Skull	2D Images	3DPs
2022	Hu	China	Spine	2D Images	2D+3DPs
2022	Lau	Australia	Heart	2D Images	2D+3DPs
2022	Nicot	France	Skull	2D Images	3DPs
2021	O’Brien	Canada	Tracheobronchus	2D Images	3DPs
2021	Karsenty	France	Heart	2D Images	2D+3DPs
2021	Tan	China	Heart	Commercial heart model	3DPs
2020	Chen	China	Gastrocolic trunk	2D Images	3DPs
2020	Tanner	United States	Skull	A half skull	3DPs
2019	Awan	United States	Hip	2D Images	2D+3DPs
2019	Bohl	United States	Spinal	2D Images	2D+3DPs
2019	Cai	Singapore	Knee	2D Images	2D+3DPs
2019	Fan	China	Bladder	2D Images	3DPs
2019	Hojo	Japan	Pelvis	Textbook	3DPs
2019	Yi	China	Skull	3D Images	3DPs
2018	Lin	China	Tuberculum sellae	2D Images	3DPs
2018	Smith	United Kingdom	Multiple organs	2D Images	3DPs
2018	Su	China	Heart	2D Images	3DPs
2018	White	United States	Heart	2D Images	2D+3DPs
2018	Wu	China	Spinal	2D Images	2D+3DPs
2017	Chen	China	Skull	Atlas	3DPs
2017	Jones	United States	Vascular rings and slings	2D Images	2D+3DPs
2017	Loke	United States	Heart	2D Images	3DPs
2017	Wang	China	Heart	Plastic model	3DPs
2016	Zheng	China	Pancreas	3D image	3DPs
2015	Li	China	Spinal	CT	3DPs
2015	Lim	Australia	Cardiac	Cadaveric materials	3DPs

**TABLE 2:** Quality of the included studies assessed by National Institute of Controlled Intervention Studies tools.

Study	Quality rating
2022_Al-Badri	Good
2022_Hu	Fair
2022_Lau	Fair
2022_Nicot	Good
2021_O’Brien	Fair
2021_Karsenty	Good
2021_Tan	Good
2020_Chen	Good
2020_Tanner	Good
2019_Awan	Fair
2019_Bohl	Fair
2019_Cai	Fair
2019_Fan	Fair
2019_Hojo	Fair
2019_Yi	Fair
2018_Lin	Good
2018_Smith	Good
2018_Su	Good
2018_White	Fair
2018_Wu	Good
2017_Chen	Fair
2017_Jones	Fair
2017_Loke	Fair
2017_Wang	Fair
2016_Zheng	Fair
2015_Li	Good
2015_Lim	Fair

### 3.2 Post-test

#### 3.2.1 3DPs and traditional tool vs. traditional tool

**FIGURE 3:**
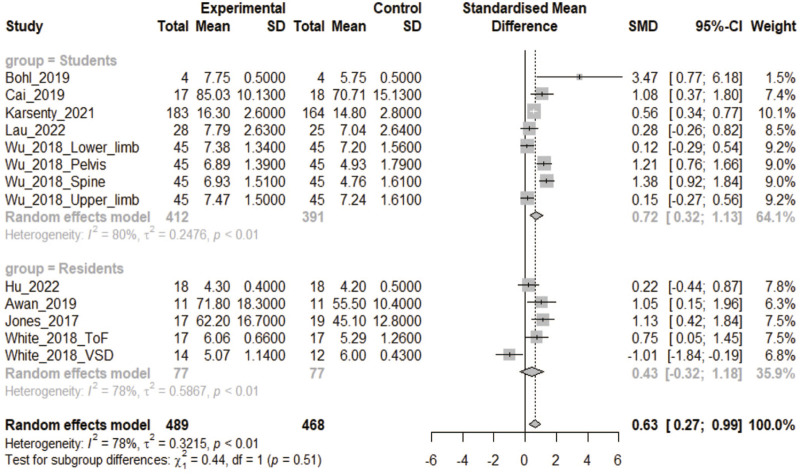
In the comparative study of 3DPs + traditional tool vs. traditional tool, the combined data were used to compare the test scores of the two groups after training. (3DPs: Three-dimensional printing models).

Nine articles including 13 CON studies were included in this analysis (Jones & Seckeler, 2017; White et al., 2018; Wu et al., 2018; Awan et al., 2019; Bohl et al., 2019; Cai et al., 2019; Karsenty et al., 2021; Hu et al., 2022; Lau & Sun, 2022). There were 489 participants in the 3DPs group and 468 in the CON group. The heterogeneity test showed that *p* < 0.1. On the whole, after training, the test results of 3DPs group were better than those of the CON group [SMD: 0.63, 95% confidence interval (CI): 0.27–0.99, *p* < 0.05; Figure 3)]. Upon dividing the analysis into subgroups, the results became more varied. For participant medical students, the combined analysis found that the 3DPs group was better than the CON group (SMD: 0.72, 95% CI: 0.32–1.13, *p* < 0.05; Figure 3). However, for residents, the combined analysis found no statistical difference between the two groups (SMD: 0.43, 95% CI: −0.32 to 1.18, *p* > 0.05; Figure 3).

#### 3.2.2 3DPs vs. 2D

**FIGURE 4:**
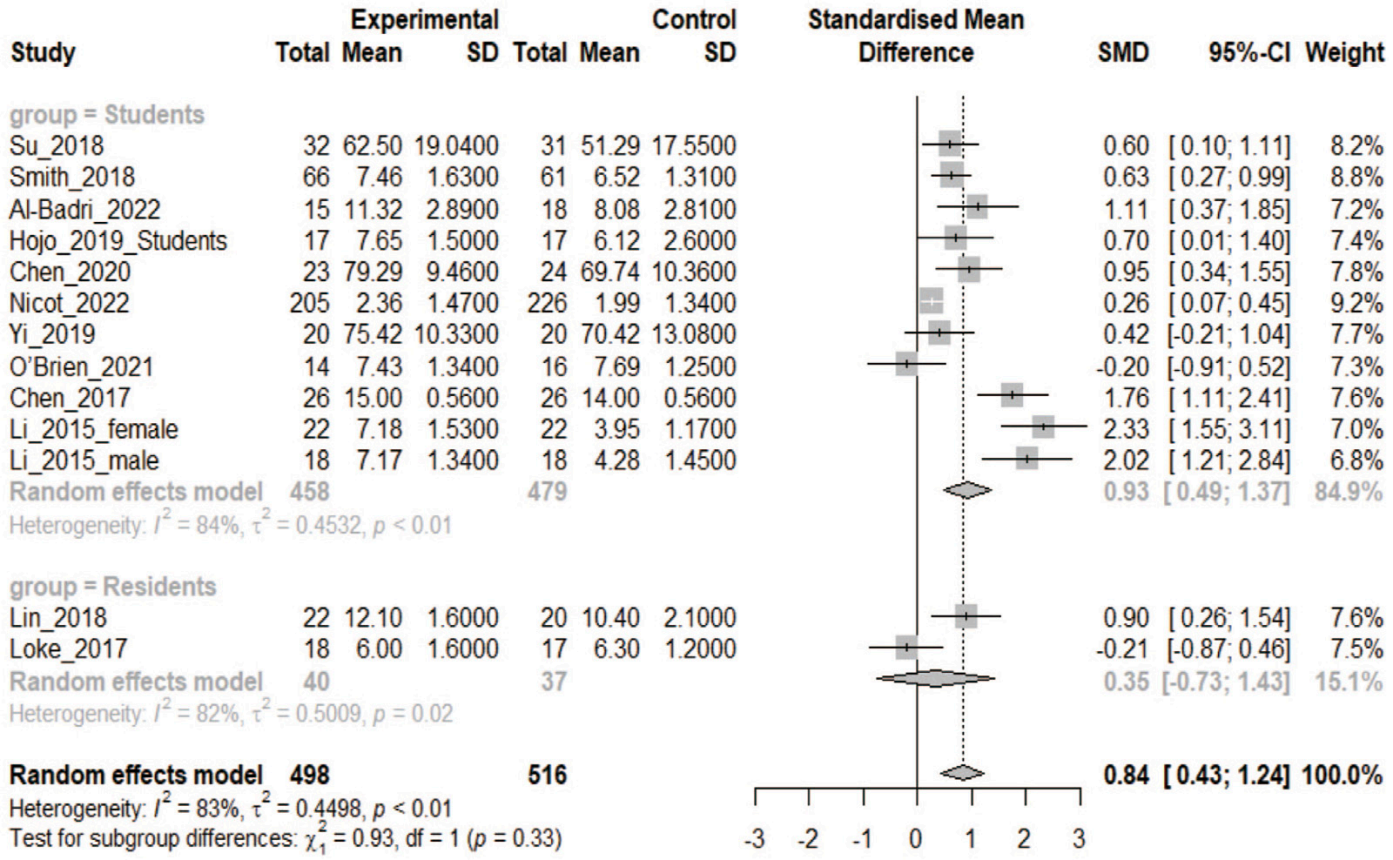
In the comparative study of 3DPs vs. 2D, the combined data were used to compare the test scores of the two groups after training. (2D: two-dimensional).

In all, 13 CON studies of 12 articles were included in this analysis (Al-Badri et al., 2022; Chen et al., 2020; Chen et al., 2017; Hojo et al., 2019; Li et al., 2015; Lin et al., 2018; Loke et al., 2017; Nicot et al., 2022; O'Brien et al., 2021; Smith et al., 2018; Su et al., 2018; Yi et al., 2019). There were 498 participants in the 3DPs group and 516 in the CON group. The heterogeneity test yielded a *p*-value <0.1. Combined data analysis found that the test scores of the 3DPs group were better than those of the CON group (SMD: 0.84, 95% CI: 0.43–1.24, *p* < 0.05; Figure 4). Subgroup analysis showed that the 3DPs group with students as participants performed better than the CON group (SMD: 0.93, 95% CI: 0.49–1.37, *p* < 0.05; Figure 4). However, there was no difference between the 3DPs group whose participants were residents and the CON group (SMD: 0.35, 95% CI: −0.73 to 1.43, *p* > 0.05; Figure 4).

#### 3.2.3 3DPs vs. 3DI

**FIGURE 5:**
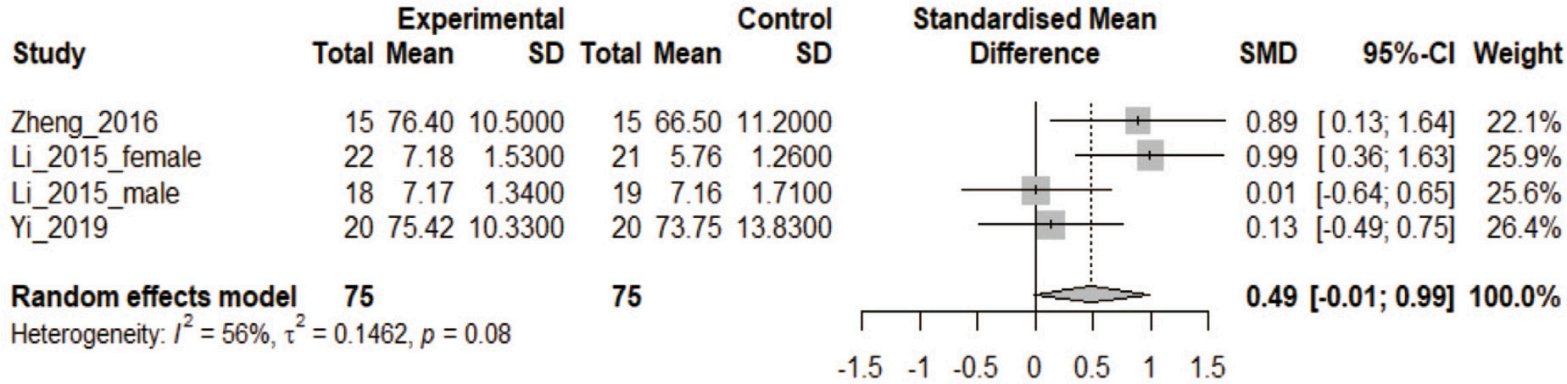
In the comparative study of 3DPs vs. 3DI, the combined data were used to compare the test scores of the two groups after training. (3DI: 3D visual imaging).

Three articles containing four CON studies were included in this analysis (Li et al., 2015; Zheng et al., 2016; Yi et al., 2019). The heterogeneity test showed that *p* < 0.1. There were 75 participants in the 3DPs group and 75 in the 3DI group. The combined data found no statistical difference in test scores between the two groups (SMD: 0.49, 95% CI: −0.01 to 0.99, *p* > 0.05; Figure 5).

#### 3.2.4 3DPs vs. physical model

**FIGURE 6:**
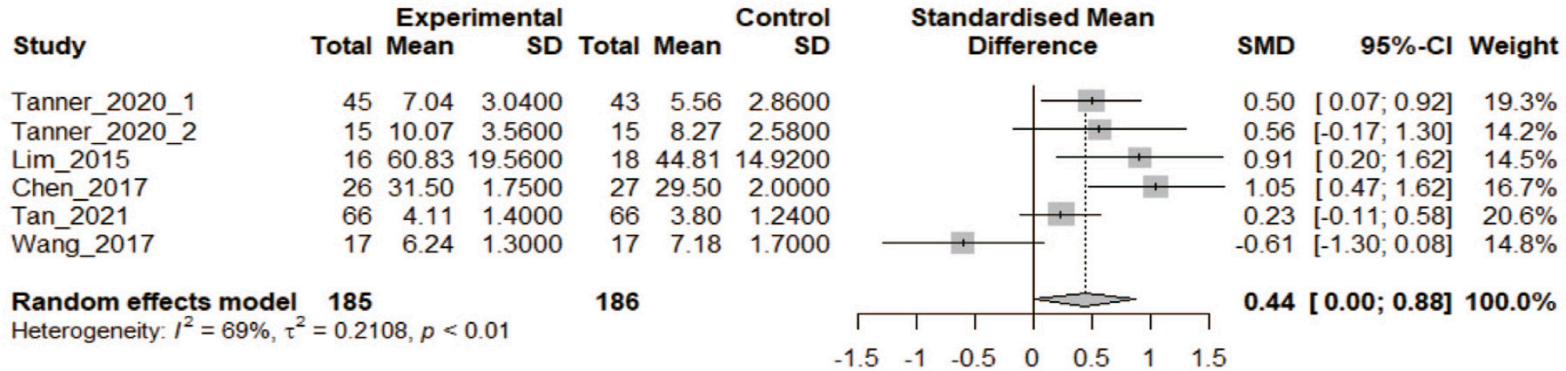
In the comparative study of 3DPs vs. Physical model, the combined data were used to compare the test scores of the two groups after training.

Five articles including six CON trials were included in this analysis (Lim et al., 2016; Chen et al., 2017; Wang et al., 2017; Tanner et al., 2020; Tan et al., 2021). There were 185 participants in the 3DPs and 186 in the CON group. The heterogeneity test yielded *p* < 0.1. Meta-analysis showed that the 3DPs group performed better on the test than the CON group (SMD: 0.44, 95% CI: 0.00–0.88, *p* < 0.05; Figure 6).

### 3.3 Satisfaction analysis

**FIGURE 7:**
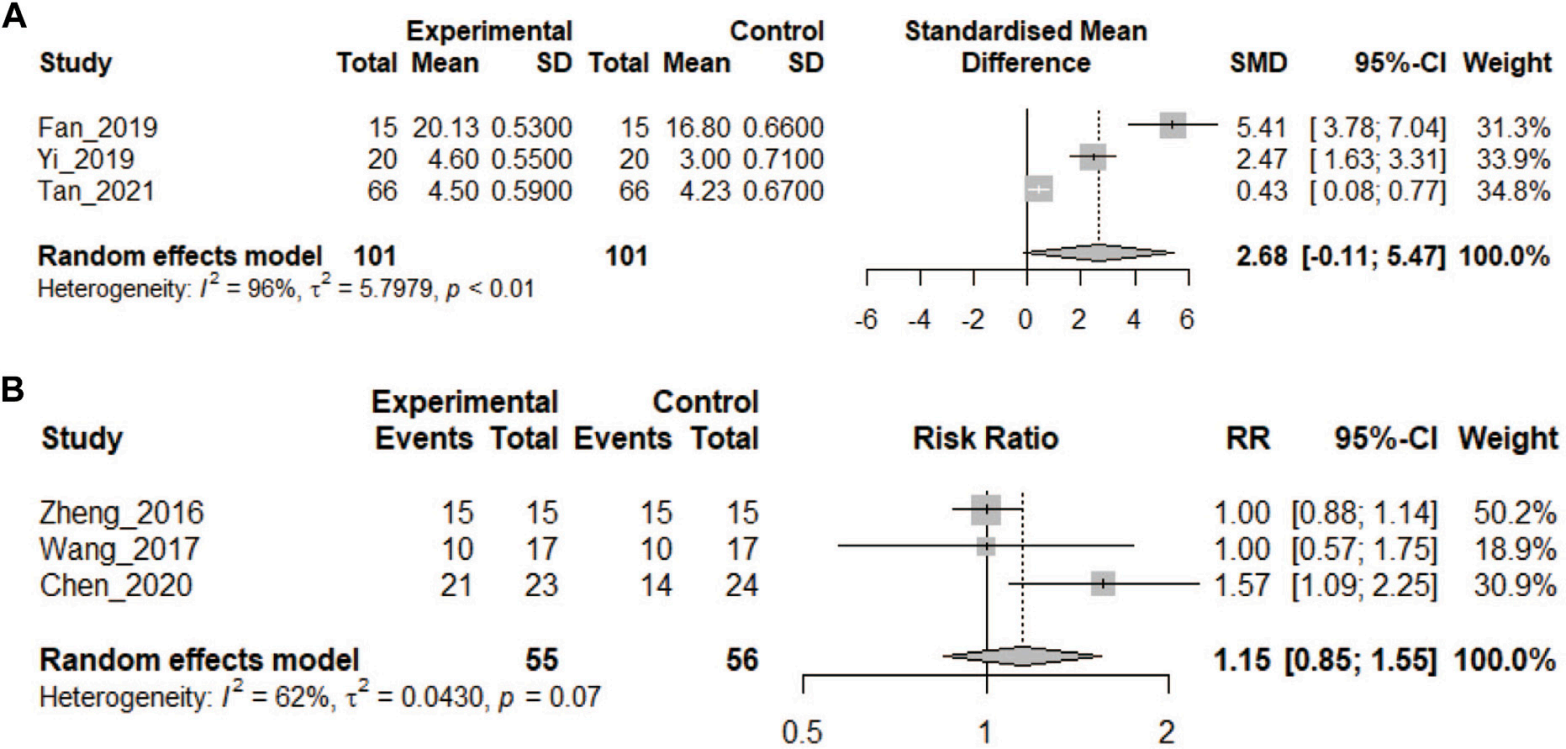
**(A)** In the comparative study of 3DPs vs. control, the continuous variable data were combined to compare the post training satisfaction of the two groups. **(B)** In the comparative study of 3DP vs. control, the binary variable data were combined to compare the satisfaction of the two groups after training.

Six articles reported satisfaction. Only one of these had residents as participants (Zheng et al., 2016); in the others they were medical students. The heterogeneity test yielded *p*-values <0.1 as well. Among them, three studies scored satisfaction, and the data were treated as continuous (Fan et al., 2019; Yi et al., 2019; Tan et al., 2021). Continuous meta-analysis of variables indicated that as for the satisfaction score of the 3DPs group vs. the CON group, the difference was not statistically significant (SMD: 2.68, 95% CI: −0.11 to 5.47, *p* = 0.06; Figure 7A).

In the other three studies, a yes/no judgment was made of satisfaction, and the data were treated as dichotomous (Zheng et al., 2016; Wang et al., 2017; Chen et al., 2020). Meta-analysis of the binary variables showed that the satisfaction rate of the 3DPs group (83.6%) vs. CON group (69.6%) was not statistically significant (RR = 1.15, 95% CI: 0.85–1.55, *p* > 0.05, Figure 7B).

### 3.4 Sensitivity analysis

**FIGURE 8:**
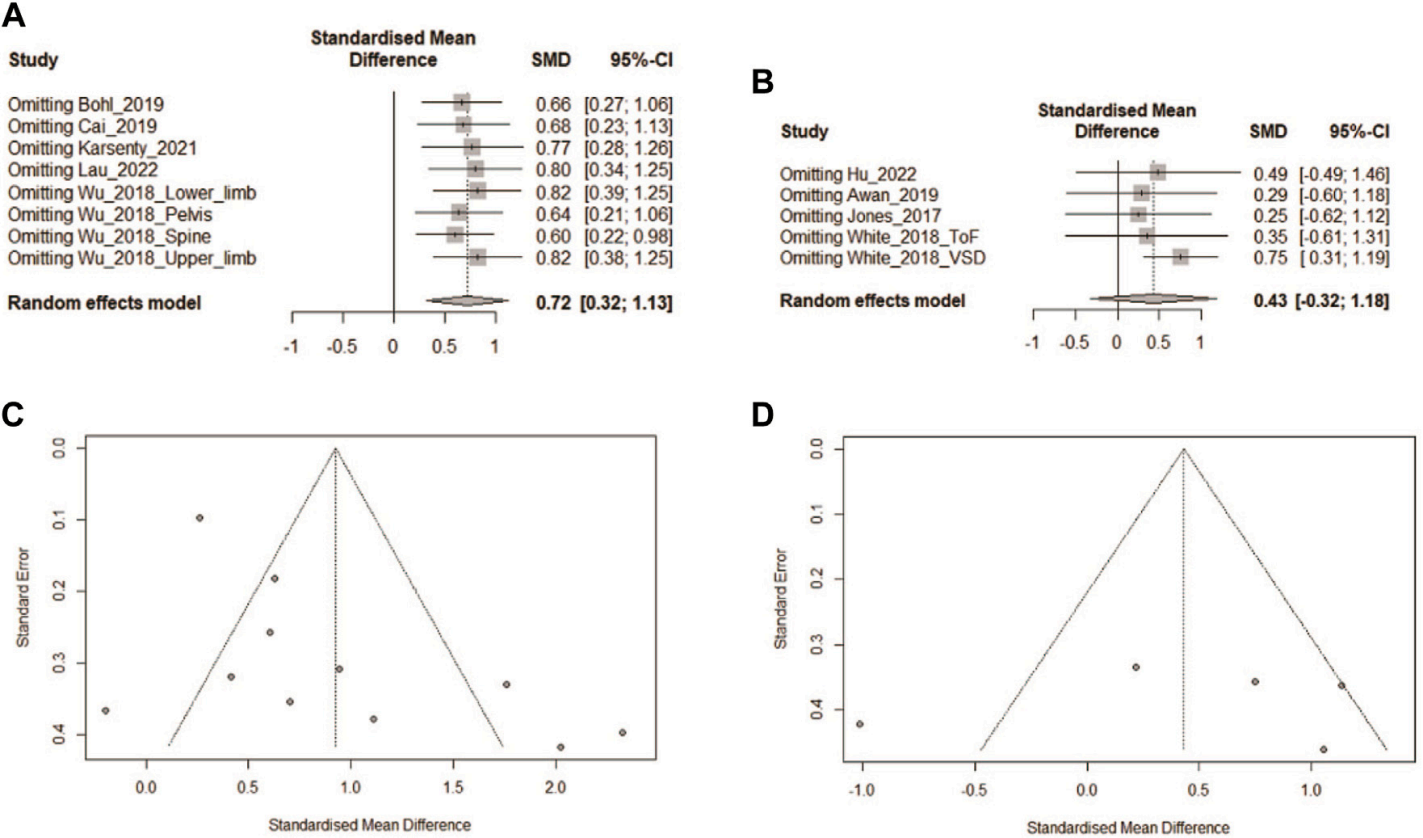
**(A)** The leave-one-out method was used to conduct an influence analysis for the subgroup of medical students in 3.2.1. **(B)** The leave-one-out method was used to conduct an influence analysis for the subgroup of medical students in 3.2.2.**(C)** Funnel chart for the student subgroup in 3.2.1. **(D)** Funnel chart for the student subgroup in 3.2.2.

The data in 3.2.1 were tested by the leave-one-out method. For CON studies with medical students, leave-one-out showed that the test performance of the 3DPs group was always better than that of the CON group (*p* < 0.05, Figures 8A,B). However, the results for the CON study of the resident group changed: When study “White_2018_VSD” was removed, the test results of the 3DPs group were better than for the CON group (*p* < 0.05). This indicates that in the resident group, the study “White_2018_VSD” influenced the results after data merging.

Sensitivity analysis was performed on the data of the medical students in 3.2.2, and the results remained stable when data from any of the studies were removed. This shows that the results have good stability and high reliability.

### 3.5 Publication bias

Publication bias tests were performed for the data in 3.2.1 for medical students and residents separately, and the Begg and Egger results were both greater than 0.05 (Figures 8C,D). However, for the data in 3.2.2 for medical students, Egger test yielded *p* < 0.05, indicating potential publication bias. Publication bias means that literature with positive results may be more likely to be published. Therefore, the results in 3.2.2 for the medical student group may require more samples for data merging, and the reliability of the current results is not good.

### 3.6 Meta-regression analysis

**FIGURE 9:**
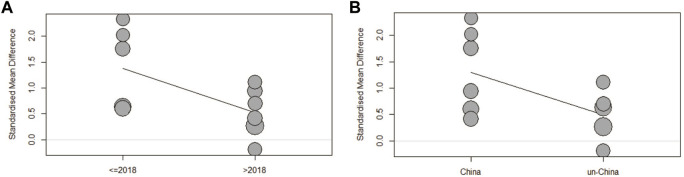
**(A)** Regression analysis of year of publication and SMD. **(B)** Regression analysis between countries of publication and SMD. (SMD: standardized mean difference).

Regression analysis was performed on the data of 3.2.2 medical students, and it was found that publication year and country affected SMD values (Figures 9A,B, *p* < 0.05). Therefore, the time and region of publication might have caused the heterogeneity. The effect of organ location and participant identity on SMD was not statistically significant. Similarly, the regression analysis of the medical student group in 3.2.1 did not find that the time, country, or organ anatomical position had statistically significant effects on SMD.

## 4 Discussion

3D printing has become a leading medical technology with highly productive and cost-effective manufacturing methods suitable for anatomical models, tissue engineering models, and medical devices and instruments (Fan et al., 2020). 3DP can be used as an auxiliary technology in teaching, and can be combined with medical anatomy, clinical surgery, and instrument inspection (Ford & Minshall, 2019). Among the published studies, the most common application of 3D printing technology reported in medicine is surgical planning, followed by medical education and training (Pietrabissa et al., 2020). The articles included in this study are mainly from China. Regarding time of publication, 2018 and 2019 were the peak years in the publication of relevant studies. Since the outbreak of COVID-19 occurred in China in early 2020, China has implemented strict CONs that have had a certain impact on the publication of teaching papers on 3DPs, so the number of articles included in this study from 2020 is small (Zhou et al., 2020). However, in general, the literature on 3DPs included in this study has increased over time since 2015. The meta-analysis included 27 articles, probably with the largest number of articles to date on the use of 3DPs in medicine.

All heterogeneity tests yielded values less than 0.1, so we used the random fixed effect model. The possible reasons for heterogeneity include the methodological diversity of the CON studies, the diversity of participants’ identities, the diversity of interventions, and so on. For example, the subjects included in the study include junior medical students, senior medical students, and residents.

In this study, we explored the role of 3DP models in human anatomy teaching. This study analyzes two important issues of concern. One is the change of test scores after training with 3DP model compared with the CON group; the other is the feedback of participants on the satisfaction of 3DP model. For the test results after training, we focused on the results for anatomical theory, excluding the results of operation training. There were two main forms of CON trials included in the study, 3DPs + CON vs. CON and 3DPs vs. CON, for which we defined special groups.

For the 3DPs + CON vs. CON trials, the test scores of the 3DPs group were significantly higher than those of the CON group, and the sensitivity analysis results remained stable among medical students. Conversely, for residents, there was no difference between the two groups. The above information shows that in medical colleges, the 3DP model for medical students can yield better test scores than in the CON group. Conversely, as residents had already learned anatomical knowledge, although their performance in the 3DPs group was higher than that in the CON group, there was no statistical difference between the two groups.

Most the CON trials were conducted in the 3DPs vs. CON mode. In this mode, the CON group used conventional teaching tools, such as 2D, 3D, and physical models. For medical students, the combined data show that the performance of the 3DPs group was better than that of 2D group, but there may be potential publication bias. Regression analysis showed that the time and country of publication affected the results. Specifically, if the article was published after 2018 or outside China, the experimental group will have smaller SMD values than the CON group. The performance of 3DPs group was better than that of physical model group, but sensitivity analysis showed that the result was unstable, which may be due to the small number of participants. Regarding the overall performance of the 3DPs group vs. the 3DI group, the difference was not statistically significant. The above results show that the test scores from using 3DPs are higher than for the CON group regardless of the identity of the participants and the teaching aids of the CON group, but there are no statistical differences found in individual subgroups, which cannot hide the good efficiency of 3DPs.

The trainers’ feedback content is extensive and varied regarding such issues as the effectiveness and practicality of the model, the help of anatomical knowledge, satisfaction, and self-confidence in learning anatomy (Wang et al., 2017; Yi et al., 2019). In the previous meta-analysis (Ye et al., 2020), because there were few studies included, we adopted the method of descriptive analysis to evaluate satisfaction. This meta-analysis included many studies, and the data were sufficient to be combined for the analysis of continuity or binary variables. Satisfaction was the generalization and refinement of the overall feedback, so the second form of content we paid attention to here was the satisfaction of participants. Interestingly, regardless of data being continuous or binary, the satisfaction of the 3DPs group showed a good satisfaction feedback, but compared to the CON group, there was no statistical difference. This indicates good acceptance of the 3DP model by students.

Sensitivity analysis shows that the removal of the “White_2018_VSD” study affects the stability of the results (Figure 8B). Perhaps increasing the sample size or increasing the CON study would yield more stable results. Regression analysis found that the time of publication and the source of the authors affected the SMD values in the 3.2.2 data. Articles published after 2018 reported smaller SMD values. A possible reason is that people have gradually come to better understand 3DPs, the novelty of 3DPs is decreasing, and the CON tests are becoming increasingly strict. These articles were from China, which means that for the larger SMD values, there might be a publication bias.

3D printing has innovative advantages, including low cost, low loss, customized geometry, and easy replacement of materials (Saroia et al., 2020). 3D printing technology is widely used in medicine, not only in anatomy teaching and clinical surgery operation training, but also in cell structure printing and skin tissue engineering, where it is expected to help treat skin diseases (Kim et al., 2018). The cost and size of 3D printers have declined over the past decade (Nicot et al., 2022).

The 3DP model also has disadvantages. Some of the challenges facing 3D printing are material selection, manufacturing time, low mechanical properties, void formation, lack of geometric stability after extrusion, and overall cost-effectiveness (Saroia et al., 2020). The bionic effect of 3DP is poor, and it is difficult to print out the organ structure with complex structure; safety and effective supervision need to be further improved; and there is a lack of ideal bioinks to solve the problem of transplant rejection (Li et al., 2022). The development of 3D printing technology is accompanied by moral challenges, such as the ethical issues of the source of printing materials and of model application (Jones, 2019). With the continuous progress of technology, under the premise of strictly abiding by medical ethics, we can expect the application of 3DPs to gradually increase in medical teaching and training and continuously contribute to the progress of medicine. With the progress of science and technology, 4D printing has appeared. 4D printing is an extension of 3D printing technology that uses traditional 3D printers to embed product design into deformable smart materials, allowing it to print objects that change over time (Shie et al., 2019). Thus, in the future 4D printing technology will also appear in medical teaching.

### 4.1 Limitations and prospects

Individual CON studies did not describe the randomization method. Some CON studies had a small number of participants, and some had a total number less than 30. The main source of articles was China, followed by the United States, accounting for 63% of the articles included. For the data in Section 3.2.2, regression analysis found that articles from China had a larger SMD value, which means that articles published in China might more easily yield positive results, describing more favorable aspects of 3DPs and fewer unfavorable ones. Owing to a limited number of randomized CON trials, we did not specifically analyze the 3D model of a particular organ. Despite the large number of 3D heart models, we do not want to repeat the analysis as we have previously examined and reported on the literature (Ye et al., 2020). After about a quarter-century of research and development, only a handful of commercial alloys can be printed, and the market value of all 3D-printed products is a tiny fraction of that of the entire manufacturing economy. This is partly due to significant differences in the structure and performance of printed components (Mukherjee & Debroy, 2019). 3DP also faces many challenges, such as the source of printed primitive organs, ethical issues, cost issues, refinement issues, and model service life issues. We look forward to the continuous progress of 3D printing technology, and expect 3DP to bring more surprises to clinical medicine.

## 5 Conclusion

Generally speaking, medical students or residents use 3DPs to learn anatomical knowledge, and score higher on tests than those of with commonly used teaching aids. In the medical student group, it was found that the 3DPs group was statistically different from the CON group, but the resident group showed no statistical difference. Medical students and residents report good satisfaction with 3DPs. 3DPs also faces many challenges, such as issues of medical ethics, printing of precision structure, and product quality. We look forward to the continuous progress of 3D printing technology for the benefit of medical anatomy education.

## Data Availability

The raw data supporting the conclusions of this article will be made available by the authors, without undue reservation.
